# Ultra-Clean Pure Shift ^1^H-NMR applied to metabolomics profiling

**DOI:** 10.1038/s41598-019-43374-5

**Published:** 2019-05-03

**Authors:** Juan M. Lopez, Rodrigo Cabrera, Helena Maruenda

**Affiliations:** Pontificia Universidad Católica del Perú, Departamento de Ciencias – Química, CERMN, Av. Universitaria 1801, Lima, 32 Peru

**Keywords:** Biophysical chemistry, Solution-state NMR

## Abstract

Even though Pure Shift NMR methods have conveniently been used in the assessment of crowded spectra, they are not commonly applied to the analysis of metabolomics data. This paper exploits the recently published SAPPHIRE-PSYCHE methodology in the context of plant metabolome. We compare single pulse, PSYCHE, and SAPPHIRE-PSYCHE spectra obtained from aqueous extracts of *Physalis peruviana* fruits. STOCSY analysis with simplified SAPPHIRE-PSYCHE spectra of six types of Cape gooseberry was carried out and the results attained compared with classical STOCSY data. PLS coefficients analysis combined with 1D-STOCSY was performed in an effort to simplify biomarker identification. Several of the most compromised proton NMR signals associated with critical constituents of the plant mixture, such as amino acids, organic acids, and sugars, were more cleanly depicted and their inter and intra correlation better reveled by the Pure Shift methods. The simplified data allowed the identification of glutamic acid, a metabolite not observed in previous studies of Cape gooseberry due to heavy overlap of its NMR signals. Overall, the results attained indicated that Ultra-Clean Pure Shift spectra increase the performance of metabolomics data analysis such as STOCSY and multivariate coefficients analysis, and therefore represent a feasible and convenient additional tool available to metabolomics.

## Introduction

Metabolomics is a state-of-the-art approach dedicated to the identification of small molecule metabolites with the aim to understand the physiological and pathological processes of complex biological systems. Mass Spectrometry (MS) and Nuclear Magnetic Resonance (NMR) spectroscopy stand out as the most powerful techniques for this purpose. Metabolites are known to be faster and more accurately quantitated, in a cost-efficient manner, without much sample manipulation by NMR^1^. High-throughput NMR profiling has been employed in different areas in agriculture^2^, agroindustry^3^, nutrition^4^, medicine^5^, biomarkers discovery^6^, pharmacology^7^, veterinary^8^, forensics^9^, chemometrics^10^, chemotaxonomy^11^, quality control^12^, denomination of origin^13^, among others.

Various chemical entities, such as peptides, nucleic acids, amino acids, organic acids, carbohydrates, phenolics, at various concentration levels and in a wide range of molecular weights (<1500 Da), are usually encountered in samples of biological origin. Although ^1^H NMR is commonly used in metabolic profiling, it has a drawback: a large number of signals with expanded multiplicities confined in a narrow spectral range can lead to extensive overlap, thereby complicating the analysis and interpretation of the spectrum^1^. To overcome this limitation, various homonuclear and heteronuclear multidimensional experiments are necessary to improve resolution and metabolite identification, all of which are time consuming, are more prone to miscalibration and pulse imperfections, and can present signals artifacts. In the case of ^1^H-^13^C HSQC, the gain in resolution is compromised by poor sensitivity of the method, due to low abundance of the heteroatom. On the other hand, Pure Shift NMR, method through which homonuclear J coupling multiplicities are collapsed into single lines^14,15^, has shown to be a convenient analytic tool to address crowded spectra. However, it has seldom been employed in proton metabolomic profiling scenarios.

In 1976, early work presented by Ernst and coworkers described the first Pure Shift experiment using a 45° projection of the 2D J resolved spectrum^16^. As signals obtained in J resolved experiments are a combination of absorption and dispersion line shapes, absolute value processing is required before the 45° projection, leading to Pure Shift spectra with broad and long tailed peaks^14,16^. Improved versions of this projected J resolved spectrum have been proposed for metabolomic studies^17^.

In 1997, Zangger and Sterk published a novel Pure Shift method where homonuclear J couplings are refocused by a combination of a broadband 180° pulse with a spatially selective 180° pulse^18^. The homonuclear decoupled spectrum is registered as a pseudo 2D in which each increment records a small portion of the FID. In each increment, the J coupling is refocused at the beginning of the block. Finally, the total FID is reconstructed using interferogram methodology^18^. Whereas this method achieved excellent homonuclear decoupling, the spatial slice selection of the signal lead to poor signal intensity (1–5%) compared to that of a regular hard pulse proton spectrum^15^.

In 2014, a novel interferogram Pure Shift method based on an anti-Z-COSY sequence^19,20^, PSYCHE (Pure Shift Yielded by Chirp Excitation)^21^, facilitated high-resolution Pure Shift spectra with a somewhat higher sensitivity (10–20% compared to that of regular proton hard pulse experiments).

It is known that chunk constructed FID experiments as PSYCHE and Zangger-Sterk suffer from periodic side band artifacts arriving from small J coupling evolution during the acquisition of each block^22^. In the analysis of pure compounds these periodic chunking artifacts can be neglected as they normally represent less than 5% of the parent peak^22^. However, in biological samples this is no longer possible, as the sideband artifacts of some metabolites could be as large, or larger, than that of dilute mixture constituents, compromising the accuracy of the metabolic profile data obtained.

Recently, Moutzouri and coworkers published a modification of the regular PSYCHE experiment - Sideband Averaging by Periodic PHase Incrementation of Residual J Evolution method (SAPPHIRE) - through which these periodic artifacts were eliminated^22^. The novel method is based on a cyclic phase modulation of chunking sidebands and their suppression by the addition of each phase increment. Consequently, SAPPHIRE-PSYCHE experiments ensure Ultra-clean Pure Shift spectra, with almost no sensitivity penalty with respect to the classical PSYCHE method^22^.

Another drawback of Pure Shift methods is the different types of artifacts generated by strongly coupled nuclei. In recent years, efforts have been made to eliminate these artifacts; nevertheless, there is still no method available to suppress all of them^14,23,24^. In the particular case of periodic artifacts due to strong coupling evolution, SAPPHIRE has shown to be quite effective in their suppression^22^.

Despite the availability of a large variety of Pure Shift NMR methods^21^, none have been used to address biological mixtures. Hence, in this paper we report the use of SAPPHIRE-PSYCHE methodology in the study of *Physalis peruviana* fruits^25^. We show that the enhanced resolution achieved by the SAPPHIRE-PSYCHE experiment combined with Statistical TOtal Correlation SpectroscopY (STOCSY)^26^ and multivariate analysis can lead to improved metabolic pattern recognition in a complex plant mixture.

## Experimental Section

### Sample preparation

Fruits belonging to *P*. *peruviana* plants grown in six different Andean regions: San Marcos, Celendin I, Celendin II, Celendin III, Bambamarca I, and Bambamarca II^25^, were extracted as described in Maruenda *et al*.^25^. Ten independent extractions (n = 10) were performed in all cases, except for Cape gooseberries from Celendin III (n = 7). NMR samples contained 200 mM sodium oxalate buffer pH 4, TSP (5 mM), and maleic acid (20 mM) as the internal quantitative NMR standard. The estimated concentrations for the previously identified metabolites fall within the 0.5 mM–110 mM range.

### NMR analyses

All NMR spectra were recorded at 20 °C on a Bruker 500 MHz, Avance III HD equipped with cryoprobe and autosampler. Flip angles were calibrated for each sample using 360° pulse optimization. Regular ^1^H NMR spectra were acquired using a 30° flip angle, 64 scans, 20 ppm spectral width, 64 K complex data points, and total relaxation time of 4.28 s. Spectra were processed with exponential apodization of 0.3 Hz. Pure Shift experiments were recorded with 2 K complex points and 5 KHz spectral width in the direct dimension. Adiabatic excitation was performed with a 20° flip angle double saltire CHIRP pulse, 30 ms duration, 10 kHz sweep-width combined with a weak field gradient of 1.08 Gauss/cm. The total relaxation time for each experiment was 3.6 s. Regular PSYCHE experiments were acquired as a pseudo 2D spectrum with 256 transients and 16 Pure Shift interferogram with 39.063 Hz spectral width. The total experimental time was 4 h 28 m. SAPPHIRE-PSYCHE experiments were acquired in a pseudo 3D manner with 32 transients, 8 SAPPHIRE interferogram in F2, and 16 Pure Shift interferogram with 39.063 Hz spectral width in F1. Since SAPPHIRE needs to compensate the duration of the first block, an extra block was systematically acquired in F1 in other to achieve this compensation^22^. The total time for this experiment was 4 h 48 m. Pure Shift spectra were reconstructed as described by Moutzouri *et al*.^22^. Final Pure Shift spectra were processed with zero filling to 32768 complex data points and apodised with a π/2 shifted sine-bell. SAPPHIRE-PSYCHE pulse sequence and automation processing programs were downloaded from Manchester NMR Methodology Group website (https://www.nmr.chemistry. manchester.ac.uk/?q=node/426). All spectra were processed using Topspin 3.5 pl 7.

### Chemometrics

Full resolution ^1^H NMR and SAPPHIRE-PSYCHE spectra were imported into MATLAB Version R2018a (Mathworks, Natick, MA) and aligned using the Icoshift tool^27^. Empty regions and those including residual water (from 4.66–5.18 ppm) and reference standards^25^ were excluded, resulting in 26877 data points for both ^1^H NMR and Pure Shift spectra. All spectra were normalized to total intensity. Statistical total correlation analysis was performed without bucketing^26,28,29^. Partial least squares-discriminant analysis was performed without bucketing^28,29^ after Pareto scaling^30^. In order to validate the multivariate model, 5-fold cross-validation was employed using the ropls package^31^ available on R statistical software.

## Results and Discussion

**Figure 1:**
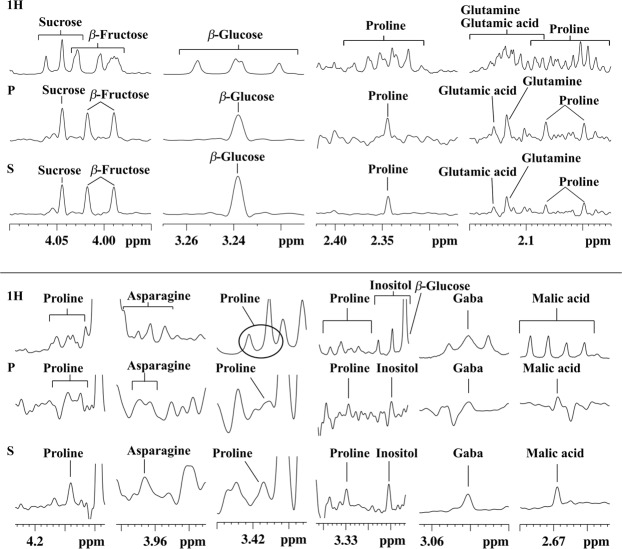
Selected expansion regions of ^1^H NMR (1H), PSYCHE (P), and SAPPHIRE (S) spectra of an aqueous extract of Cape gooseberry (Bambamarca I) showing signal assignments.

In our previous work^25^, twenty-three (23) compounds were thoroughly identified and quantified by NMR in aqueous extracts of Cape gooseberry. Extensive overlap between proton signals (see full spectrum in Supplementary Fig. S1) complicated the assessment. Various of these crowded ^1^H NMR spectrum regions have been expanded and shown in Fig. 1. Suppression of J couplings by PSYCHE, as expected and shown in the expanded area, Fig. 1 (P) (top), improved signal resolution for sucrose (4.04 ppm), *β*-fructose (4.02 ppm and 3.99 ppm), *β*-glucose (3.24 ppm), proline (2.34 ppm, 2.07 ppm, and 2.00 ppm), glutamic acid (2.16 ppm), and glutamine (2.13 ppm). The gain achieved in the latter three signals is noteworthy. These experiments allowed us to unravel glutamine from glutamic acid. The presence of the latter was impossible to confirm in the early study using classical NMR experiments. Through the STOCSY analysis presented in next section, other glutamic acid proton NMR signals were depicted and by HSQC (data not shown), all carbon correlations were later confirmed. As expected with PSYCHE, the appearance of J modulated interferogram artifacts compromised the assignment, particularly in the case of less intense and highly overlapped signals: proline (4.13 ppm, 3.41 ppm, and 3.33 ppm), asparagine (3.97 ppm), myo-inositol (3.27 ppm), GABA (3.03 ppm), and malic acid (2.67 ppm), Fig. 1 (P) (bottom). In all cases, SAPPHIRE-PSYCHE removed these artifacts and allowed a clearer identification of the signals, Fig. 1 (S). As expected, the SAPPHIRE sequence does not completely eliminate the strong coupling artifacts. Some remain for glutamic acid (2.16 ppm, Fig. 1), glutamine (2.13 ppm, Fig. 1) and citric acid (2.75 ppm and 2.87 ppm, Supplementary Fig. S2).

To evaluate the extent to which Pure shift methodology affects quantitation, several non-overlapped signals in the ^1^H NMR spectrum (Supplementary Table S1) were selected for the necessary comparisons. The error attained varied between 11 to 34%, as shown in Table S1. This result was expected. SAPPHIRE-PSYCHE is a pulse sequence comprised of more than a single pulse, therefore it is susceptible to magnetization loss due to relaxation and diffusion between defocused and refocused pulse field gradients. Removal of the zero-quantum and cross-peak magnetization coherence components of the signal caused by the double saltire swept pulse in the presence of a weak field gradient^21^, in addition to signal truncation and recoupling artifacts^21^, are also factors to consider in order to explain the differences attained. In this study, no attempts have been made to improve the quantitative nature of the Pure Shift pulse sequence. However, as demonstrated elsewhere with HSQC^32,33^, time zero spectrum methods combined with SAPPHIRE-PSYCHE may be a feasible approach for this purpose.

### Statistical total correlation spectroscopy

Statistical TOCSY is a useful tool to depict resonances of nuclei which belong to the same metabolite in complex ^1^H NMR profiles. The method is based on the calculation of the correlation matrix between each NMR signal resulting in an pseudo homonuclear correlation spectrum where cross peaks represent the correlation values^26^. The method takes advantage of the collinearity existing among signal intensities of nuclei within the same molecule. These correlations are also observed with molecules linked through metabolic pathways^26^. However, the latter are normally not as intense as intramolecular correlations.

**Figure 2:**
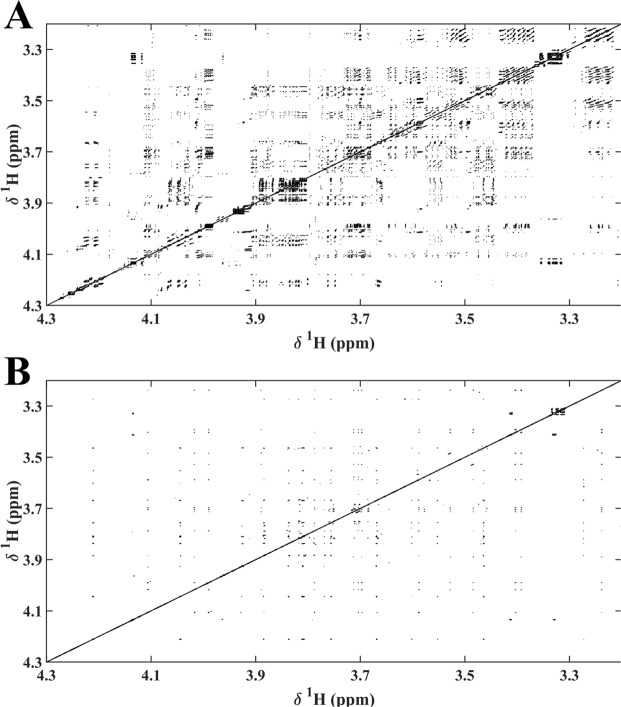
Selected expanded regions (3.20 ppm–4.30 ppm) of two-dimensional STOCSY NMR spectra obtained with data from six Cape gooseberry extracts showing correlation values (r^2^) above 0.85: (**A**) 2D STOCSY of ^1^H NMR spectra and (**B**) 2D STOCSY of SAPPHIRE-PSYCHE spectra.

The use of classical ^1^H NMR STOCSY in the study of complex mixtures is limited by unavoidable signal overlap, which in some cases can lead to complete loss of correlations. It is also affected by the complexity of the correlation pattern, where the strong existent correlations between peaks comprising J-coupled signals play a determinant role. Ultra-clean Pure Shift method, by converting J coupling multiplicity correlations into a single correlation peak and thereby reducing overlapping, is expected to simplify the STOCSY correlation matrix. This is here demonstrated by comparing the ^1^H NMR STOCSY and SAPPHIRE-PSYCHE STOCSY correlation matrices obtained with Cape gooseberry extracts, Fig. 2. As shown, Ultra-clean Pure Shift method increased STOCSY resolution and allowed a clearer identification of cross correlation peaks.

**Figure 3:**
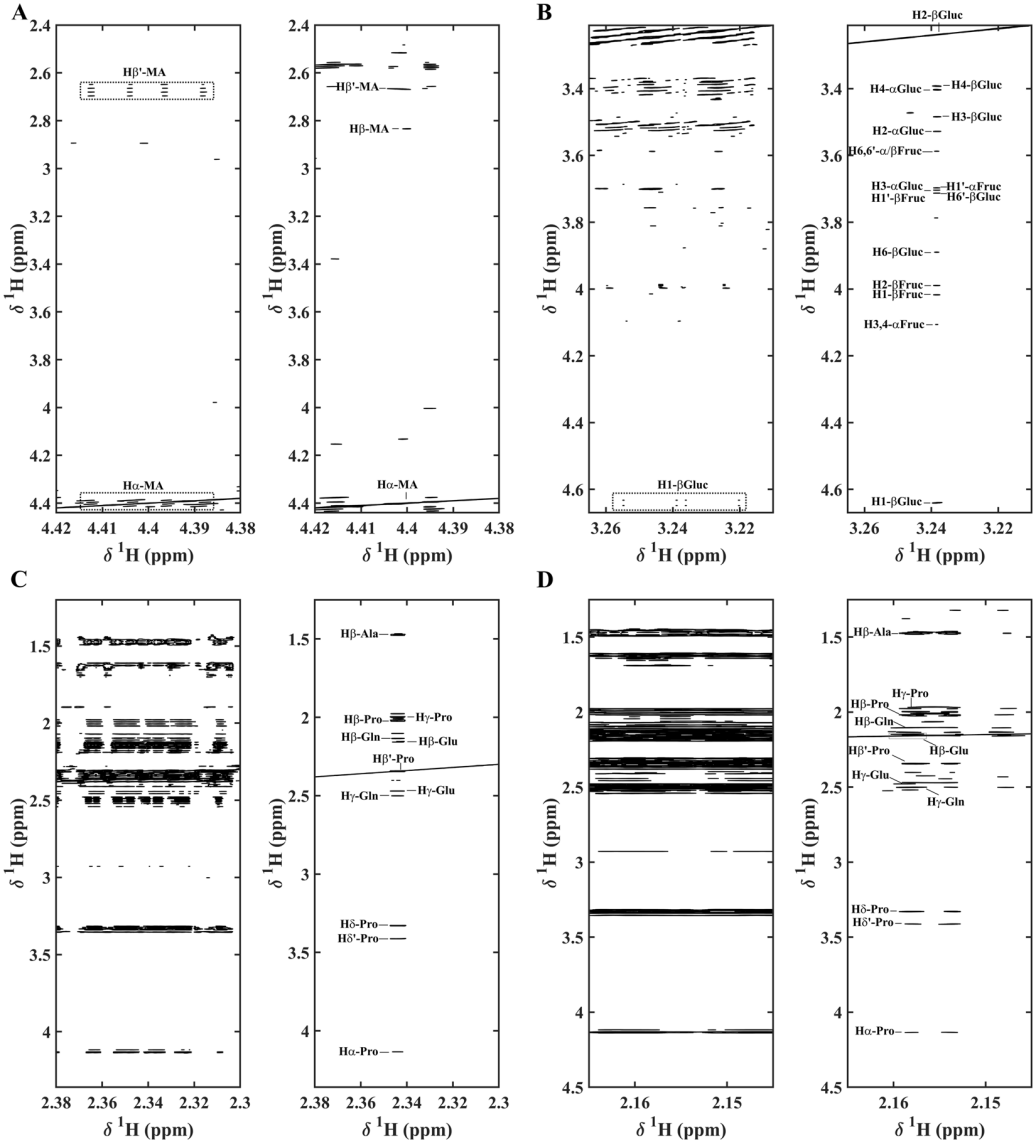
Two-dimensional STOCSY representations of NMR spectra of six different Cape gooseberry extracts showing correlations (r^2^), in the left without homodecoupling and in the right with homodecoupling for regions: (**A**) 4.38–4.42 ppm and 2.40–4.42 ppm with r^2^ above 0.80 for malic acid (MA) signal (H*α*-MA); (**B**) 3.21–3.27 ppm and 3.21–4.67 ppm with r^2^ above 0.85 for *β*-glucose signal (H2-β-Gluc); (**C**) 2.30–2.38 ppm and 1.25–4.36 with r^2^ above 0.93 for proline (Pro) signal (Hβ′-Pro); (**D**) 2.15–2.17 ppm and 1.25–4.5 ppm with r^2^ above 0.90 for glutamic acid (Glu) signal (Hβ-Glu); *α*-glucose, *α*-fructose, *β*-fructose are symbolized as *α-*Gluc, *α-*Fruc, and *β-*Fruc, respectively.

Several regions of the 2D spectra have been expanded in Fig. 3 to better visualize the magnitude of the gain achieved by using SAPPHIRE-PSYCHE STOCSY. The complex multiplet patterns associated with the nuclei Hα (4.41 ppm), Hβ (2.82 ppm), and Hβ′ (2.67 ppm) of malic acid (Fig. 3A) are shown to collapse cleanly into discrete cross-peaks. Correlation peaks from *β*-glucose, obscured by the presence of signals associated with *α*-glucose, *α*-fructose, and *β*-fructose - all of which strongly correlate with each other given that they are linked to the same metabolic pathways- are more clearly recognized in SAPPHIRE-PSYCHE STOCSY, Fig. 3B. Other strongly correlated metabolites in Cape gooseberry were found to be the amino acids^25^. However, their identification in crowded regions of the spectrum was a difficult task. With 2D Ultra-clean Pure Shift STOCSY (Fig. 3C), proline intermolecular correlations: Hβ′-Hα, Hβ′-Hβ, Hβ′-Hγ, Hβ′-Hδ, and Hβ′-Hδ′ and proline intramolecular correlations with alanine, glutamine, and glutamic acid are depicted. Moreover, the simplified 2D NMR spectra attained allowed the corroboration of the other proton signals Hβ (2.13 ppm) of the newly identified glutamic acid, through its correlation with Hγ (2.44 ppm) (Fig. 3D).

**Figure 4:**
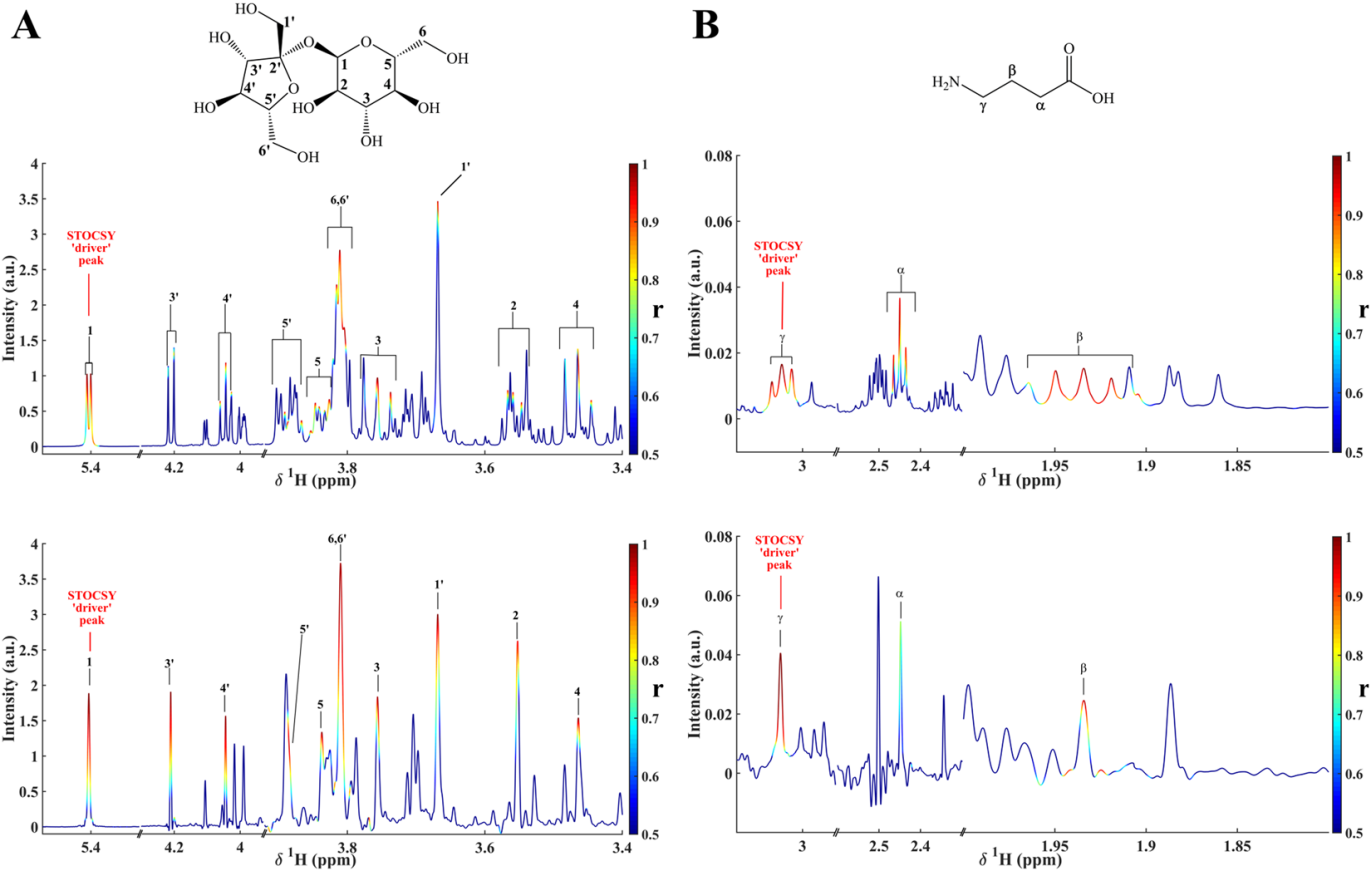
Comparison between one-dimensional STOCSY obtained with the ^1^H NMR spectra (top) and the SAPPHIRE-PSYCHE spectra (bottom) of Cape gooseberry samples. Correlation coefficients (r) have been color coded and projected on the average of all spectra: (**A**) 1D STOCSY obtained from sucrose signal at 5.40 ppm (STOCSY driver peak); (**B**) 1D STOCSY obtained from GABA signal at 3.03 ppm (STOCSY driver peak).

The STOCSY analysis was simplified, as suggested by Cloarec *et al*.^26^, by extracting a single correlation vector at a given resonance (driver peak) from the whole correlation matrix. This correlation vector, plotted as color code on the NMR spectrum, generates monodimensional STOCSY spectra (1D STOCSY)^26^. In Fig. 4, the 1D STOCSY correlations obtained from classical ^1^H NMR and SAPPHIRE-PSYCHE experiments, for sucrose and GABA, are shown. While GABA is easily determined by either STOCSY method, this is not the case for sucrose, for which extensive signal overlap decreases the intramolecular correlation and complicates the identification of the nuclei of interest (Fig. 4B). With Pure Shift STOCSY, all sucrose resonances, even H-5′ signal partially overlapped with H-6 from *α*-glucose and H-3 from β-fructose, were better discriminated.

### Chemometrics

**Figure 5:**
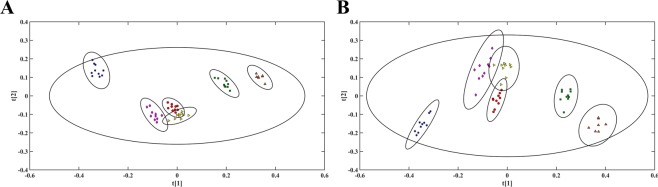
PLS scores of Cape gooseberries extracts grown in six different Andean regions (San Marcos: red circles, Celendin III: brown triangles, Bambamarca I: blue stars, Celendin I: yellow triangles, Bambamarca II: green squares, Celendin II: magenta diamonds) constructed based on (**A**) classical ^1^H NMR and (**B**) SAPPHIRE-PSYCHE experiments. Hotelling’s T^2^ ellipses were set to 95% confidence level.

Multivariate analyses, PCA (Principal component analysis) and PLS-DA (Partial least squares-discriminant analysis), were performed with ^1^H NMR and Ultra-clean Pure Shift NMR spectra. The discrimination performance was slightly affected by the homonuclear decoupling. Worth to mention is the better separation along PC2 achieved with the Pure Shift method for the samples San Marcos and Celendin I (Supplementary Fig. S3). From the PLS score plots, Fig. 5, it is clear that the SAPPHIRE-PSYCHE model displays higher score group standard deviations along t2 when compared to the regular ^1^H NMR model (Supplementary Table S2). This observation may be due to lower signal to noise ratios in Pure Shift spectra. The accuracy of both PLS-DA models, assessed through the total explained variation of X and Y (R^2^X, R^2^Y) and cross-validation factor (Q^2^) values (Table 1), indicate similar discrimination efficiency.

**Table 1:** Discrimination accuracy between ^1^H NMR and SAPPHIRE-PSYCHE PLS-DA of Cape gooseberries data.

Component	^1^H NMR			SAPPHIRE-PSYCHE		
	R^2^X	R^2^Y	Q^2^	R^2^X	R^2^Y	Q^2^
1	0.348	0.192	0.187	0.377	0.192	0.188
2	0.089	0.192	0.221	0.129	0.177	0.206
3	0.079	0.173	0.187	0.072	0.184	0.228

R^2^X = Total explained variation of X.

R^2^; Y = Total explained variation of Y.

Q^2^ = cross-validation factor.

**Figure 6:**
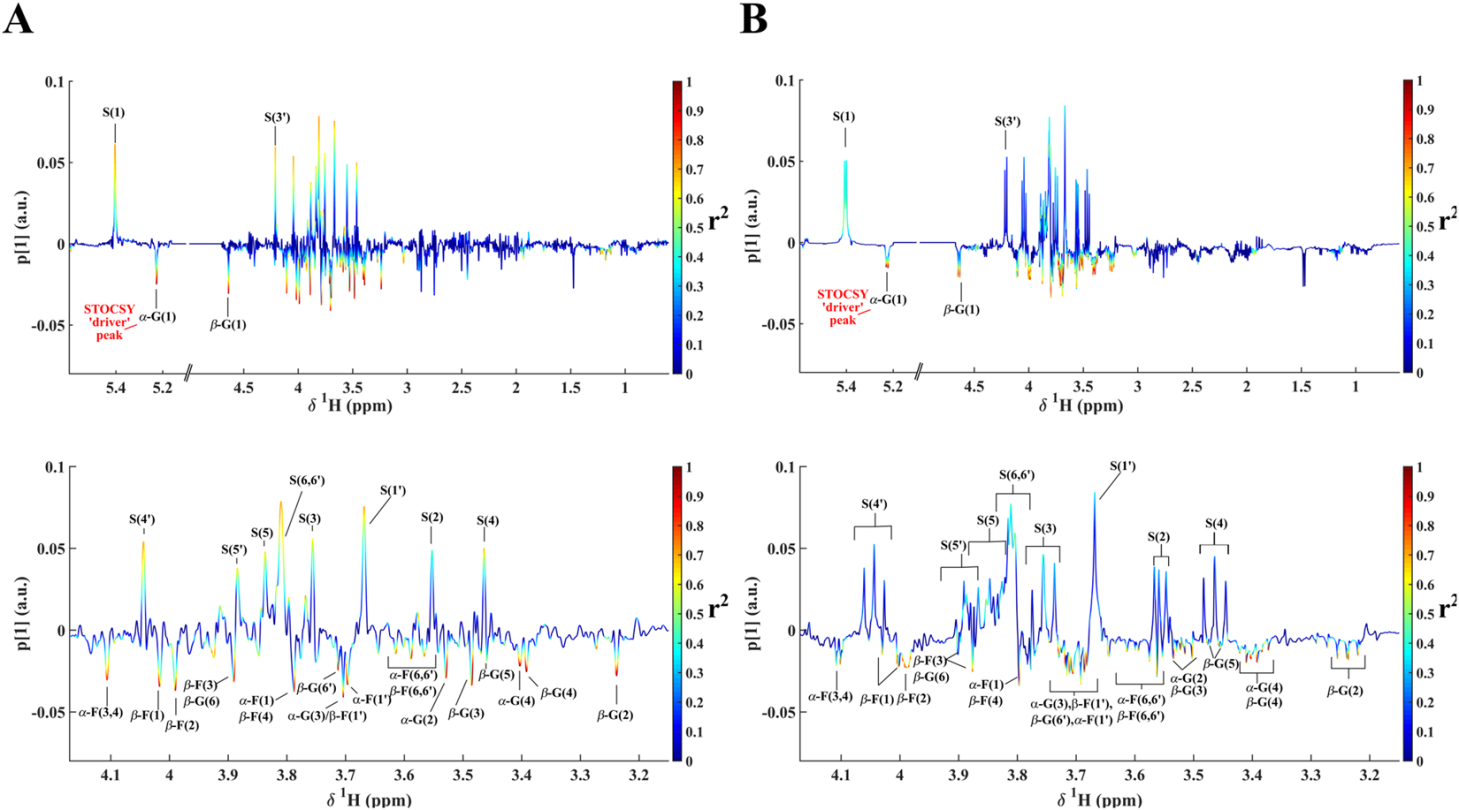
Combination of PLS1 loadings and 1D STOCSY for *α*-glucose correlation using the STOCSY signal at 5.23 ppm as driver peak. The coefficient of determinations (r^2^) have been color coded and projected on the coefficients of the first PLS component: (**A**) 1D STOCSY obtained with SAPPHIRE-PSYCHE data (top) and its expansion 3.15–4.17 ppm (bottom); (**B**) 1D STOCSY obtained with ^1^H NMR data (top) and its expansion 3.15–4.17 ppm (bottom); *α*-glucose, *β*-glucose, *α*-fructose, *β*-fructose, and sucrose are symbolized as *α*-G, *β*-G, *α*-F, *β*-F, and S, respectively.

The clear improvement of the Pure Shift model in the multivariable analyses is evident when inspecting the loadings. The increased resolution obtained by applying SAPPHIRE-PSYCHE simplified the analysis of the PLS-DA loadings and allowed better distinction of the specific contribution of each sugar signal (*α*-glucose, *β*-glucose, *α*-fructose, *β*-fructose, and sucrose) to sample discrimination, as shown in Fig. 6.

Moreover, Cloarec *et al*. showed that PLS coefficients combined with 1D STOCSY enhances biomarkers identification^26,34^. Using this approach, the plot of *α*-glucose Pure Shift STOCSY on the first PLS orthogonal projection (Fig. 6A, bottom), allowed us to clearly define the strong correlation existent among *α*-glucose (driver peak), *β*-glucose, *α*-fructose, and *β*-fructose, and their anti-correlation with sucrose. With classical STOCSY analysis (Fig. 6B, bottom), the extensive spectral overlap diminishes the correlation coefficients, and leads to loss of important metabolic pathway information.

## Conclusion

This paper applied modern Pure Shift proton spectroscopy in a plant metabolomic study examining aqueous extracts of *P*. *peruviana* fruits harvested from six different Andean ecosystems. The enhanced capabilities of SAPPHIRE-PSYCHE methodologies allowed easier identification of metabolites present in the complex mixture and led to the identification of an additional metabolite that escaped the previous analysis by standard proton NMR. 2D STOCSY obtained with SAPPHIRE-PSYCHE spectra is more straightforward to analyze and could more easily be used with NMR databases for structure identification based on the chemical shift^35^. Ultra-clean Pure Shift spectra, hence, can increase the performance of metabolomics data analysis such as statistical TOCSY and multivariate coefficients analysis.

## Supplementary information

Supplementary Information: Ultra-Clean Pure Shift 1H-NMR applied to metabolomics profiling
