# Supplementary material for: Ultra-Clean Pure Shift 1H-NMR applied to metabolomics profiling
Source: Sci Rep. 2019 May 3;9:6900. doi: 10.1038/s41598-019-43374-5 (PMC6499883; doi:10.1038/s41598-019-43374-5)
Supplement: Supplementary file 1 — Supplementary Information: Ultra-Clean Pure Shift 1H-NMR applied to metabolomics profiling [file 41598_2019_43374_MOESM1_ESM.pdf]

Supplementary Information

Ultra-Clean Pure Shift ^{1}H-NMR applied to metabolomics profiling

Juan M. Lopez[1], Rodrigo Cabrera[1], Helena Maruenda[1].

[1]Pontificia Universidad Católica del Perú, Departamento de Ciencias – Química, CERMN, Av. Universitaria 1801, Lima 32, Perú

Table S1. Concentrations (mean ± standard deviation) (mg/g dry weight) of selected compounds in aqueous extracts of Bambamarca I obtained from ^{1}H NMR and SAPPHIRE-PSYCHE spectra. In brackets the Chemical shift in ppm of the signal used for quantification.

Metabolite (δ in ppm)	^{1}H NMR	SAPPHIRE-PSYCHE	Error (%)
Alanine (1.48)	3.98±0.04	4.63±0.19	14.04
Choline (3.19)	0.26±0.02	0.32±0.02	18.45
α-Fructose (4.11)	29.42±0.63	33.70±1.45	12.70
β-Fructose (3.99)	98.45±1.97	148.49±7.47	33.70
GABA (3.03)	2.67±0.05	3.49±0.40	23.48
α-Glucose (5.23)	45.16±0.58	61.14±1.56	26.13
β-Glucose (4.64)	77.70±1.27	108.54±4.08	28.41
Myo-inositol (3.28)	12.28±0.79	11.09±1.83	10.73
Malic acid (4.41)	8.22±0.20	9.33±0.45	11.85
Sucrose (5.41)	222.68±5.43	329.01±9.24	32.32
Valine (1.04)	0.65±0.01	0.50±0.02	29.26

Table S2. PLS scores standard deviation for each group of Cape gooseberries extracts grown in six different Andean regions.

Cape gooseberry	1H-NMR		SAPPHIRE-PSYCHE	
	t[1]	t[2]	t[1]	t[2]
Bambamarca I	0.017	0.031	0.021	0.042
Bambamarca II	0.019	0.024	0.015	0.036
Celendin I	0.022	0.015	0.019	0.038
Celendin II	0.017	0.028	0.026	0.068
Celendin III	0.014	0.018	0.021	0.030
San Marcos	0.014	0.017	0.013	0.037

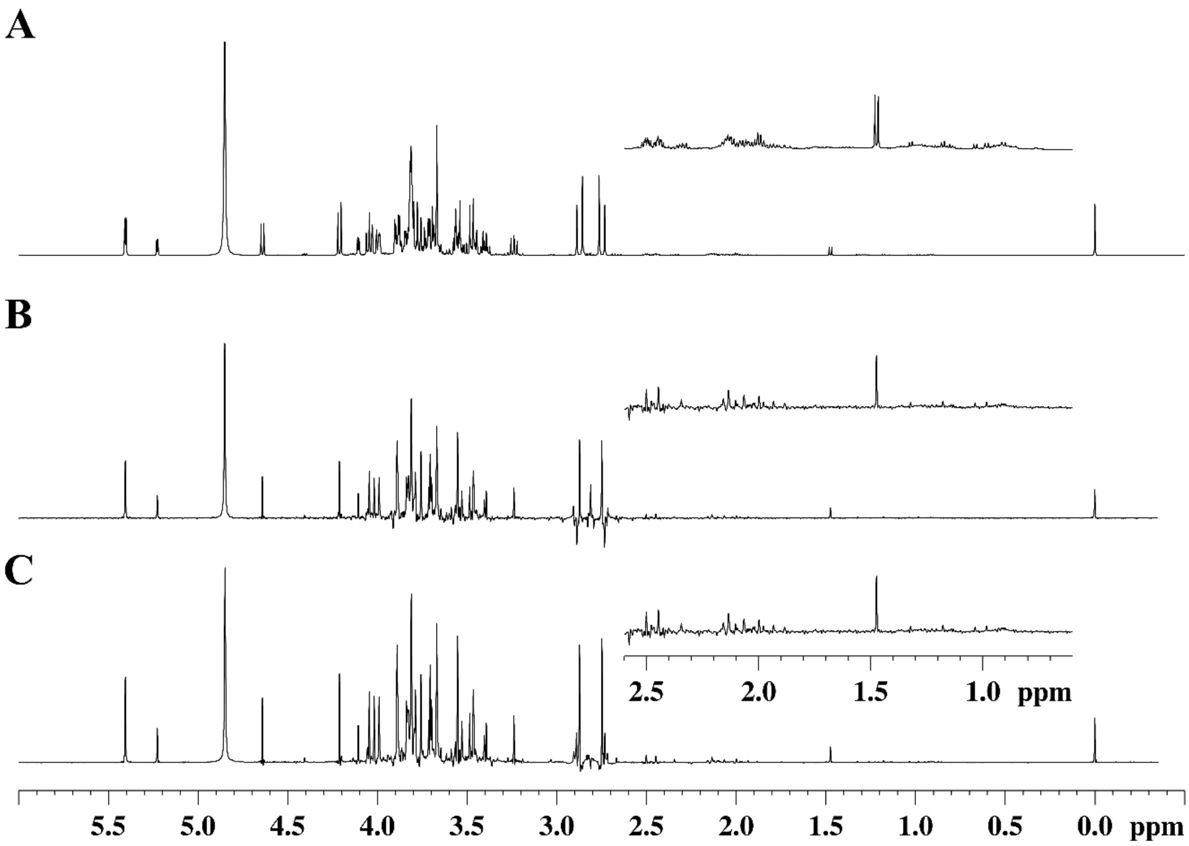

Figure S1. Representative (A) ^{1}H NMR, (B) PSYCHE, and (C) SAPPHIRE-PSYCHE spectra of an aqueous extract of Cape gooseberries (Bambamarca I)

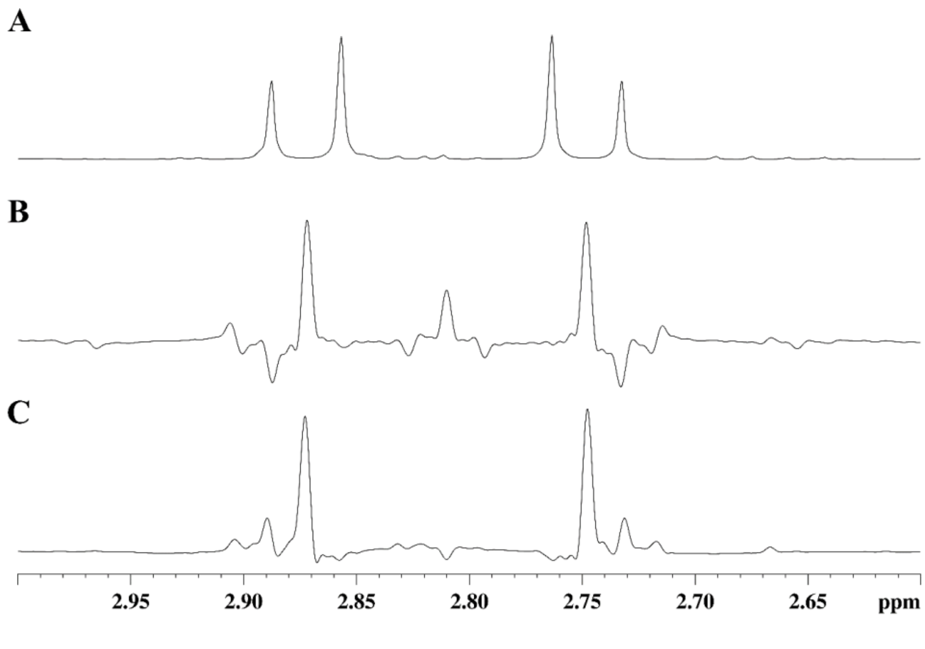

Figure S2. Expansion (2.60-3.00 ppm) of representative (A) ^{1}H NMR, (B) PSYCHE, and (C) SAPPHIRE-PSYCHE of an aqueous extract of Cape gooseberries (Bambamarca I) showing strongly coupled signals from citric acid.

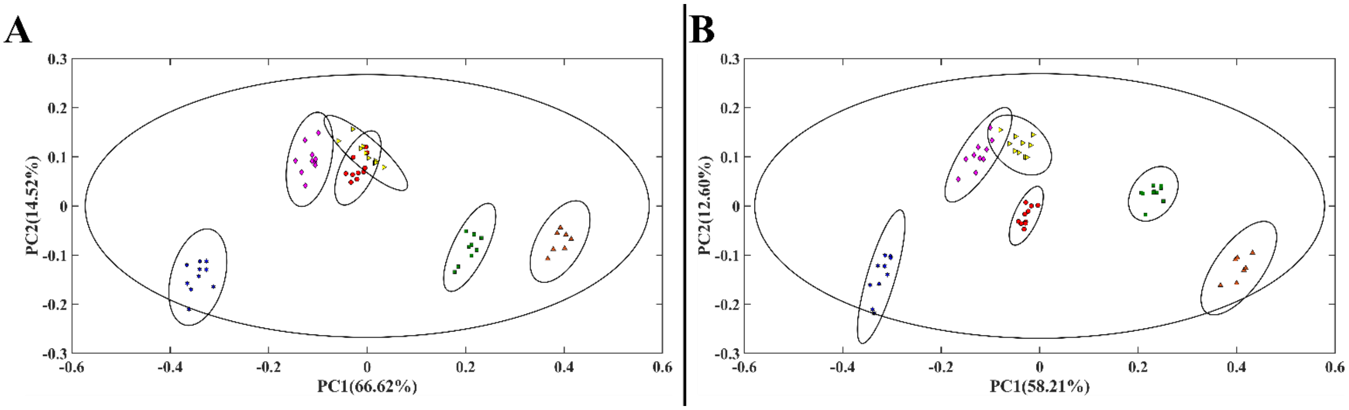

Figure S3. PCA scores of Cape gooseberries extracts grown in six different Andean regions (San Marcos ●, Celendin III ▲, Bambamarca I ✱, Celendin I ▷, Bambamarca II ■, Celendin II ◆) constructed based on A) classical ^{1}H NMR and B) SAPPHIRE-PSYCHE experiments. Analysis was performed with 0.009 ppm bucketing and Pareto scaling, hotelling´s T^2 ellipses were set to 95% confidence level.
